# Chronic kidney disease of unknown aetiology in Sri Lanka: is cadmium a likely cause?

**DOI:** 10.1186/1471-2369-12-32

**Published:** 2011-07-05

**Authors:** Kamani P Wanigasuriya, Roshini J Peiris-John, Rajitha Wickremasinghe

**Affiliations:** 1 Department of Medicine, University of Sri Jayewardenepura, Nugegoda, Sri Lanka; 2 Department of Physiology, University of Sri Jayewardenepura, Nugegoda, Sri Lanka; 3 Section of Epidemiology and Biostatistics, University of Auckland, Auckland, New Zealand; 4 Department of Public health, University of Kelaniya, Ragama, Sri Lanka

**Keywords:** Chronic kidney disease, aetiology, cadmium

## Abstract

**Background:**

The rising prevalence of chronic kidney disease (CKD) and subsequent end stage renal failure necessitating renal replacement therapy has profound consequences for affected individuals and health care resources. This community based study was conducted to identify potential predictors of microalbuminuria in a randomly selected sample of adults from the North Central Province (NCP) of Sri Lanka, where the burden of CKD is pronounced and the underlying cause still unknown.

**Methods:**

Exposures to possible risk factors were determined in randomly recruited subjects (425 females and 461 males) from selected areas of the NCP of Sri Lanka using an interviewer administered questionnaire. Sulphosalicylic acid and the Light Dependent Resister microalbumin gel filtration method was used for initial screening for microalbuminuria and reconfirmed by the *Micral *strip test.

**Results:**

Microalbumnuria was detected in 6.1% of the females and 8.5% of the males. Smoking (p < 0.001), alcohol use (p = 0.003), hypertension (p < 0.001), diabetes (p < 0.001), urinary tract infection (UTI) (p = 0.034) and consumption of water from wells in the fields (p = 0.025) were associated with microalbuminuria. In the binary logistic regression analysis, hypertension, diabetes mellitus, UTI, drinking well water in the fields, smoking and pesticide spraying were found to be significant predictors of microalbuminuria.

**Conclusions:**

Hypertension, diabetes mellitus, UTI, and smoking are known risk factors for microalbuminuria. The association between microalbuminuria and consumption of well water suggests an environmental aetiology to CKD in NCP. The causative agent is yet to be identified. Investigations for cadmium as a potential causative agent needs to be initiated.

## Background

There is an increasing trend in mortality and morbidity due to chronic kidney disease (CKD) in Sri Lanka. Although epidemiological data are lacking at present, this burden is even more pronounced in the North Central Province (NCP) of Sri Lanka where the underlying cause of CKD remains unrecognized [1].

The NCP was the seat of Sri Lanka's ancient kingdoms and the centre of civilization and Buddhism. The province extends over 10 530 m^3 ^in the dry zone of the country and has a sophisticated irrigation system of water storage reservoirs. Despite its unique history and culture, NCP has been a fertile ground for infectious diseases such as malaria and Japanese encephalitis in the past. CKD is a major health problem in the province at present.

There may be unrecognized environmental toxins or occupational exposures giving rise to CKD in the region, but this remains unproven. Chronic kidney disease of uncertain aetiology (CKDu) in this region mainly affects males from poor socio-economic backgrounds who are involved in paddy farming raising the possibility of agrochemical exposure as a cause for CKDu. Renal biopsy studies revealing a tubulo-interstitial nephritis with minimum inflammation and early fibrosis in patients with early CKDu also favours a common aetiological factor and possibly a toxin mediated renal disease [2]. Recent work done by us indicates an environmental aetiology. In the case-control study, being a farmer (p < 0.001), using pesticides (p < 0.001), drinking well water (p < 0.001), having a family history of renal dysfunction (p = 0.001), use of ayurvedic treatment (p < 0.001) and a history of snake bite (p < 0.001) were found to be risk factors for CKDu [3]. The present study examines these risk factors further in the community setting through detection of microalbuminuria, an early indicator of renal damage.

## Methods

Anuradhapura, the main district in the NCP of Sri Lanka, is subdivided in to 25 divisional secretariat areas. The district population was 745 693 according to the last census in 2001. Based on statistics from the Anuradhapura Teaching Hospital, subjects were selected from three divisional secretary areas namely, Medawachchiya and Padaviya which had the highest reported cases of CKDu and Rajanganaya which had a relatively low incidence of CKDu.

From each divisional secretary area, 3 grama-niladari divisions, the smallest administrative units in the country that typically have approximately 200-250 households and a population of 800-1000, were randomly selected. From each grama-niladari division, 60 households were randomly selected from the voters register. All adults over 18 years were requested to participate in the study and attend the special clinic conducted in each area. Of the total 1110 adults in the selected households, 886 participated in the study.

Blood pressure measurements were taken using standard procedures and a physical examination conducted on all subjects by a physician. A structured pre-tested interviewer-administered questionnaire was used to obtain personal data and information on risk factors and past medical history. The farmers were questioned further to find out details regarding the use of pesticides.

A spot urine sample was collected and urinary microalbumin was estimated by precipitating with 25% sulphosalicylic acid using the Light Dependent Resister micro-albumin gel filtration method. This test detects albumin concentrations in the range of 10-200 mg/L with 83% sensitivity and 73% specificity [4]. Positive results were reconfirmed for microalbuminuria by the *Micral *strip test (Roche diagnostics) in an early morning urine sample. Those who were found to have positive results for both were considered as having microalbuminuria.

Data were double entered and analyzed using SPSS (Statistical Products and Service Solutions, Chicago, USA) version 13.0. Associations between variables were tested using independent sample t-tests, chi square tests, Fisher's exact tests and Odds ratios. Ninety-five percent confidence intervals were calculated for the Odds ratios. Binary logistic regression analysis was done to control for confounding factors.

Ethical clearance was obtained from the Ethical Review Committee of the Faculty of Medical Sciences, University of Sri Jayewardenepura. Written informed consent was obtained using an approved consent form. All participants with microalbuminuria were referred to medical clinics conducted at Teaching Hospital, Anuradhapura or Medawachchiya hospital.

## Results

The sample comprised 425 females and 461 males. The males were significantly older (p = 0.016), taller (p < 0.001) and heavier (p < 0.001) as compared to the females (Table 1).

**Table 1:** Demographic characteristics of sample population

Variable	Females (n = 425)	Males (n = 461)	p-value
Mean (SD) age (years)	30.53 (20.60)	33.63 (17.4)	0.016

Mean (SD) height (cm)	62.68 (75.15)	81.90 (81.23)	< 0.001

Mean (SD) weight (kg)	25.68 (24.87)	37.09 (24.81)	< 0.001

Number (%) by occupation			

Agriculture based	302 (71.1)	393 (85.2)	0.001

Other	123 (28.9)	68 (14.8)	< 0.001

Number (%) involved in pesticide spraying	45 (14.8)	321 (81.7)	< 0.001

Number (%) by source of drinking water at home			

Well	388 (93.9)	425 (93.0)	0.572

Stream	9 (2.2)	19 (4.2)	0.099

Pipe	24 (5.8)	19 (4.2)	0.261

Tube well	17 (4.1)	22 (4.8)	0.619

Other	0 (0.0)	1 (0.2)	0.525

Number (%) by source of drinking water in the field			

From home	252 (79.7)	286 (69.9)	0.003

Well in the field	105 (33.2)	187 (45.7)	0.001

Other	19 (6.0)	36 (8.8)	0.160

A significantly higher percentage of males were involved in agriculture (p = 0.001) and pesticide spray activities (p < 0.001) as compared to the females. The source of drinking water at home was similar in the females and the males. However, females used water from home in the field more often than the males (p = 0.003). A higher percentage of males consumed water from wells in the fields as compared to the females (p = 0.001).

Micro-albuminuria was detected in 6.1% of the females (n = 26) and 8.5% of the males (n = 39) who participated in the study. A significantly higher percentage of subjects with microalbuminuria had previously smoked (p < 0.001), consumed alcohol (p = 0.003), previously been treated for hypertension (p < 0.001), diabetes (p < 0.001) and urinary tract infection (UTI) (p = 0.034) and consumed water from wells situated in fields (p = 0.025) as compared to those with no microalbuminuria (Table 2).

**Table 2:** Exposure to possible risk factors and the presence of albuminuria

Variable		Number (%) of subjects with				Odds ratio (95% CI)	**p-value**^**a**^
				
		no albuminuria _n_		albuminuria _n_			
Farmers	Yes	814	630 (77.4)	63	52 (82.5)	1.381	0.344
							
	No		184 (22.6)		11 (17.5)	(0.706-2.701)	

Involved in pesticide spraying	Yes	812	330 (40.6)	61	25 (41.0)	1.014	0.958
							
	No		482 (59.4)		36 (59.0)	(0.598-1.722)	

History of pesticide poisoning	Yes	813	38 (4.7)	63	2 (3.2)	0.669	0.439^b^
							
	No		775 (95.3)		61 (96.8)	(0.158-2.838)	

History of smoking	Yes	813	176 (21.6)	63	29 (46.0)	3.087	< 0.001
							
	No		637 (78.4)		34 (54.0)	(1.830-5.207)	

History of alcohol use	Yes	814	220 (27.0)	63	28 (44.4)	3.087	0.003
							
	No		594 (73.0)		35 (55.6)	(1.830-5.207)	

Treated for Hypertension	Yes	813	114 (14.0)	63	27 (42.9)	2.160	< 0.001
							
	No		699 (86.0)		36 (57.1)	(1.284-3.635)	

Treated for Diabetes Mellitus	Yes	812	41 (5.0)	63	13 (20.6)	4.889	< 0.001
							
	No		771 (95.0)		50 (79.4)	(2.461-9.712)	

Acute Glomerular Nephritis	Yes	814	2(0.2)	62	0 (0.0)	0.929	0.863^b^
							
	No		812 (99.8)		62 (100.0)	(0.912-0.946)	

Renal stones	Yes	814	32 (3.9)	63	1 (1.6)	0.394	0.298^b^
							
	No		782 (96.1)		62 (98.4)	(0.053-2.933)	

Urinary Tract Infection	Yes	814	199 (24.4)	63	8 (12.7)	0.450	0.034
							
	No		615 (75.6)		55 (87.3)	(0.211-0.960)	

Chronic drug use	Yes	813	43 (5.3)	62	5 (8.1)	1.571	0.248^b^
							
	No		770 (94.7)		57 (91.9)	(0.599-4.120)	

Use of Ayurvedic Treatment	Yes	814	551 (67.7)	27	45 (71.4)	1.193	0.540
							
	No		263 (32.3)		18 (28.6)	(0.678-2.102)	

Snake bite in the past	Yes	813	95 (11.7)	63	10 (15.9)	1.426	0.324
							
	No		718 (88.3)		53 (84.1)	(0.702-2.897)	

Drinking water source							

a) at home							

Well	Yes	799	732 (91.6)	63	61 (96.8)	2.792	0.102^b^
							
	No		67 (8.4)		2 (3.2)	(0.668-1.671)	

Stream	Yes	799	27 (3.4)	63	0 (0.0)	0.925	0.125^b^
							
	No		772 (96.6)		63 (100.0)	(0.907-0.943)	

Pipe borne	Yes	799	40 (5.0)	63	2 (3.2)	0.622	0.393^b^
							
	No		759 (95.0)		61 (6.8)	(0.147-2.636)	

Tube well	Yes	799	35 (4.4)	63	2 (3.2)	0.716	0.483^b^
							
	No		764 (95.6)		61 (96.8)	(0.168-3.047)	

Other	Yes	799	1 (0.1)	63	0 (0.0)	0.927	0.927^b^
							
	No		798 (99.9)		63 (100.0)	(0.910-0.944)	

b) in the field							
From home	Yes	814	496 (60.9)	63	37 (58.7)	0.912	0.730
							
	No		318 (39.1)		26 (41.3)	(0.542-1.536)	

Well	Yes	814	251 (30.8)	63	28 (44.4)	1.794	0.025
							
	No		563 (69.2)		35 (55.6)	(1.068-3.014)	

Other	Yes	814	51 (6.3)	63	1 (1.6)	0.241	0.097^b^
							
	No		763 (93.7)		62 (98.4)	(0.033-1.776)	

^a^Chi square test;

^b^Fishers exact test

In the binary logistic regression analysis the significant predictors of microalbuminuria were history of hypertension, diabetes mellitus, UTI, drinking well water in the fields, history of smoking and involvement of pesticide spraying (Table 3). Subjects with a history of hypertension, diabetes mellitus and smoking were over three-and-a half times more likely to have microalbuminuria as compared to those who had no such history. Subjects who drank well water in the field were approximately twice as more likely to have microalbuminuria as compared to those who did not. Significantly, pesticide spraying was found to be protective.

**Table 3:** Summary results of logistic regression analysis (both male and female participants included)

Variable	Regression coefficient	p-value	Odds Ratio	95% confidence interval of odds ratio
Intercept	-3.492			

History of hypertension^a^	1.539	< 0.001	4.658	2.578 - 8.414

History of Diabetes mellitus^b^	1.343	0.001	3.832	1.735 - 8.465

History of urinary tract infection^c^	-0.706	0.098	0.493	0.214 - 1.138

Drinking well water at the field^d^	0.650	0.037	1.916	1.041 - 3.527

History of smoking^e^	1.638	< 0.001	5.147	2.568 - 10.314

Involved in pesticide spraying^f^	-0.835	0.024	0.434	0.210 - 0.898

^a ^Reference category is no history of hypertension.

^b ^Reference category is no history of diabetes mellitus.

^c ^Reference category is no history of urinary tract infection.

^d ^Reference category is not using drinking water from a well in the field.

^e ^Reference category is no history of smoking.

^f ^Reference category is not involved in pesticide spraying.

## Discussion

Diabetes mellitus, hypertension, smoking, past history of urinary tract infections, pesticide spraying and consumption of water from a well situated in the field were significant predictors of microalbuminuria in the study population.

Estimation of microalbuminuria is recommended as a screening test to detect patients at an early stage of CKD. Currently, microalbuminuria is defined as urinary albumin excretion between 20-200 mg/g in men and 30-300 mg/g in women, using the urinary albumin-to-creatinine ratio in a random urine sample; 30-300 mg/24 h, if measured in a 24 h urine collection; or 20-200 micrograms/min, if measured in a timed (e.g. 4 h or overnight) urine collection [5]. A urinary albumin concentration below these limits is considered as normal excretion. The sulphosalicylic acid test was used as a cost effective screening test to detect microalbuminuria and the *micral test *was used for confirmation. Microalbuminuria is an independent predictor for end stage renal disease in patients with diabetes mellitus or hypertension and, is used as an index of renal involvement. In this study, microalbuminuria was detected in 6.1% of the females and 8.5% of the males. Similar findings were reported from USA in which microalbuminuria was detected in 6.1% of men and 9.7% of women in the general population [6]. In 19 848 elderly South-East Asians, proteinuria (defined as ≥1+ protein on urine dipstick analysis) was found in 8.5% of previously undetected Singaporeans, after excluding the 1.1% who had pre-existing renal disease [7]. In the Third National Health and Nutrition Examination Survey conducted in the USA, the prevalence of microalbuminuria was 28.8% in persons with previously diagnosed diabetes, 16.0% in those with hypertension, and 5.1% in those without diabetes, hypertension, cardiovascular disease, or elevated serum creatinine levels [6]. The authors concluded that microalbuminuria is common, even among persons without diabetes or hypertension.

Although diabetes mellitus and hypertension are known to cause CKD, it is likely that these conditions are associated with CKD in some populations, rather than being its cause. The high prevalence of microalbuminuria and its strong association with diabetes mellitus and hypertension in areas of high prevalence of CKDu investigated by us may be due to the involvement of a common aetiological factor which may be implicated in the occurrence of CKDu, hypertension and an increased susceptibility to renal damage in diabetes mellitus.

Deficiencies or excesses of trace element compounds in water or soil is known to have an impact on the health of the inhabitants of the terrain. This is particularly evident in certain areas of the dry zone of Sri Lanka where a high prevalence of dental fluorosis is found due to the high fluoride content in ground water [8] and a high prevalence of endemic goitre is found in the wet zone due to iodine deficiency [9]. Reservoirs are the main source of irrigation for paddy lands in the NCP of Sri Lanka while drinking water in the rural areas is obtained mainly from wells. Other investigators have found high levels of Cd, Fe and Pb in five reservoirs in the same study area [10]. Dissolved Cd in reservoir water and sediment was 0.03 to 0.06 mg/L and 1.78 mg/L, respectively. This is much higher than the Maximum Contaminant Level Goal of 0.005 mg/L or 5 ppb recommended by the US Environment Protection Agency. Cd content in lotus rhizomes was 253.82 mg/kg. The Provisional Tolerable Weekly intake of Cd, based on the extreme exposure by rice and fish, was also found to be high in the region [10]. Rice, the staple food, fish and lotus rhizomes are frequently consumed by the local community. In Southeast China, a major source of cadmium exposure in the general population is the consumption of cereals, particularly rice, vegetables and shellfish [11].

In this study, subjects with a history of smoking were five times more likely to have microalbuminuria as compared to those who had no such history. It is known that smoking increases the risk of nephropathy in diabetic individuals. The Multiple Risk Factor Intervention Trial found an increased risk for end-stage renal disease in smokers as compared to non-smokers which was independent of age, ethnicity, income, blood pressure, diabetes mellitus or a history of ischaemic heart disease [12].

From the findings of this study, it may be postulated that ground water in the fields and cigarettes (and possibly certain food items consumed by this population), are likely sources of an environmental toxin. There is an urgent need for studies to identify the toxin for effective interventions to be tested. Although we cannot implicate a particular toxin based on findings of the present study we postulate that Cd may well be the toxin in question and propose that further work be done to test this hypothesis. Nephrotoxicity in humans caused by high level exposure to Cd is known. Itai-itai disease following mass Cd poisoning due to contaminated water in Toyama Prefecture, Japan is documented [13,14]. In the next section, we present reasons for generating this hypothesis.

### Cd as a likely cause of CKDu

This study reconfirms previous findings of drinking water from wells situated in the field being a significant predictor of early CKDu [3]. The wells situated in the fields could have high concentrations of Cd and other toxic compounds than wells situated in home gardens, due to the extensive use of pesticides and fertilizers in paddy cultivation in the study area, a possible source of Cd. Whether fertilizers and pesticides used in this primarily agricultural setting are the sources of Cd in soil and reservoirs is not known. The concentrations of Cd and other heavy metals in different inorganic fertilizers and in pesticides have been evaluated together with the contribution of these metals in soils from their use in rice farming areas in Spain [15]. Fertilizer (superphosphate) and the three pesticides analyzed contained high amounts of Cd. A study on the potential impact of heavy metals on groundwater as a result of fertilizer use suggests significant potential groundwater pollution from Cd, Se, Mo and U caused as a result of long-term use of phosphate fertilizers. The possibility for Cd reaching the groundwater system through soil-compartments is indicated. Poor de-silting of reservoirs in NCP could lead to progressive rise in Cd levels in water which in turn feed the paddy cultivation [16]. It is also possible that high levels of cadmium could be concentrated in well water in polluted locations. In addition, farmers clean contaminated spraying equipment near the field well after spraying pesticides. Heavy metal concentrations in well water in the study area, however, are yet to be estimated.

In a review that focused on studies of the prevalence of Cd-related kidney dysfunction among population groups residing in Cd contaminated areas in China, dose-response relationships are shown between urinary Cd and the prevalence of increased levels of biomarkers of renal damage. Biomarkers of renal tubular dysfunction such as urinary beta-2-microglobulin or N-acetyl-beta-D-glucosaminidase and of glomerular kidney dysfunction (urinary albumin) were found to be associated with Cd exposure [17]. The factors that influenced these dose-response relationships included metallothionein mRNA levels, increased levels of auto antibodies in blood/plasma against metallothionein and Type II diabetes.

Increased susceptibility to Cd-induced renal damage in diabetes has been shown in population groups in China and Australia [18,19]. Diabetes mellitus may increase the risk of Cd-induced kidney damage. Metallothionein plays a critical role in protecting animals and humans against Cd-induced nephrotoxictiy [20-22]. The presence of metallothionein antibody (MT-Ab) increases the susceptibility for tubular damage among Cd workers. In persons with type II diabetes, increased levels of auto antibodies against metallothionein in blood plasma was observed at urinary Cd levels around 1 microgram/g creatinine [18]. The authors claim that the presence of MT-Ab can potentiate tubular dysfunction among diabetic subjects and that patients with high MT-Ab levels are more prone to development of tubular damage. Whether MT-Abs is found in the current study population is not known. If Cd exposure is found to be significant in the study area, susceptibility for renal damage is more likely in the presence of MT-Ab. Moreover, it is possible that diabetes mellitus in the study population may increase the risk of Cd-induced kidney damage as reported previously. These postulations need to be investigated before a definitive cause of CKDu can be established in the NCP of Sri Lanka. A recent review on Cd, diabetes and CKD concludes that Cd may be a factor in the development of some types of diabetes and the authors raise the possibility that Cd and diabetes-related hyperglycaemia may act synergistically to damage the kidney [23].

Further, cigarette tobacco is known to contain appreciable amounts of cadmium with the accumulation of cadmium in smokers being related to the number of pack-years smoked [24]. Studies have demonstrated early low-level cadmium exposure in smokers to have nephrotoxic effect on both the glomeruli and tubules [25].

In the present study the apparent protective effect of pesticide exposure on the occurrence of microalbuminuria was an unexpected finding. It is likely that some confounding factors may have contributed towards this unexpected finding. Traditionally pesticide spraying is carried out by young healthy adults whereas microalbuminuria in diabetes and hypertension are generally seen in older age groups. It is also possible that early disease may have resulted in a healthy worker effect whereby only the healthy persons may have been involved pesticide spray activities.

## Conclusions

Hypertension, diabetes mellitus, UTI, and smoking are known risk factors for microalbuminuria. Apart from these risk factors microalbuminuria is associated with drinking well water from agricultural fields in the NCP of Sri Lanka. Although it is not possible to draw firm causal conclusions from this study due to the cross-sectional nature of it, the findings are helpful in directing future longitudinal and interventional studies that are urgently needed to establish definitive causality and identify relevant protective measures. We propose further work be done to test Cd as a possible toxin in the occurrence of CKDu in the NCP of Sri Lanka.

## Abbreviations

CKD: Chronic Kidney Disease; NCP: North Central Province; CKDu: Chronic Kidney Disease of uncertain aetiology; UTI: Urinary Tract Infection; MT-Ab: Metallothionein Antibody

## Competing interests

The authors declare that they have no competing interests.

## Authors' contributions

KPW conceived of the study, participated in the design and coordination, carried out the data collection and helped draft the manuscript. RJPJ participated in the design of the study, performed the statistical analysis and drafted the manuscript. ARW participated in the design of the study, performed the statistical analysis and helped draft the manuscript. All authors read and approved the final manuscript.

## Pre-publication history

The pre-publication history for this paper can be accessed here:

http://www.biomedcentral.com/1471-2369/12/32/prepub
